# Reducing the Strength: a mixed methods evaluation of alcohol retailers’ willingness to voluntarily reduce the availability of low cost, high strength beers and ciders in two UK local authorities

**DOI:** 10.1186/s12889-016-3117-7

**Published:** 2016-05-26

**Authors:** Colin Sumpter, Elizabeth McGill, Esther Dickie, Enes Champo, Ester Romeri, Matt Egan

**Affiliations:** Camden and Islington Public Health, 222 Upper Street, London, N1 1XR England; National Institute for Health Research School for Public Health Research (NIHR SPHR), London School of Hygiene and Tropical Medicine, 15-17 Tavistock Place, London, WC1H 9SH England

**Keywords:** Evaluation, Mixed methods, Alcohol, Availability, Local policy

## Abstract

**Background:**

Reducing the Strength is an increasingly popular intervention in which local authorities ask retailers to stop selling ‘super-strength’ beers and ciders. The intervention cannot affect alcohol availability, nor consumption, unless retailers participate. In this paper, we ask whether and why retailers choose or refuse to self-impose restrictions on alcohol sales in this way.

**Methods:**

Mixed method assessment of retailers’ participation in Reducing the Strength in two London (UK) local authorities. Compliance rates and the cheapest available unit of alcohol at each store were assessed. Qualitative interviews with retailer managers and staff (*n* = 39) explored attitudes towards the intervention and perceptions of its impacts.

**Results:**

Shops selling super-strength across both areas fell from 78 to 25 (18 % of all off-licences). The median price of the cheapest unit of alcohol available across all retailers increased from £0.29 to £0.33 and in shops that participated in Reducing the Strength it rose from £0.33 to £0.43. The project received a mixed response from retailers. Retailers said they participated to deter disruptive customers, reduce neighbourhood disruptions and to maintain a good relationship with the local authority. Reducing the Strength participants and non-participants expressed concern about its perceived financial impact due to customers shopping elsewhere for super-strength. Some felt that customers’ ability to circumvent the intervention would limit its effectiveness and that a larger scale compulsory approach would be more effective.

**Conclusions:**

Reducing the Strength can achieve high rates of voluntary compliance, reduce availability of super-strength and raise the price of the cheapest available unit of alcohol in participating shops. Questions remain over the extent to which voluntary interventions of this type can achieve wider social or health goals if non-participating shops attract customers from those who participate.

## Background

In an effort to tackle the perceived negative impact of super-strength alcohol in the United Kingdom (UK), a number of local licensing authorities have encouraged ‘Reducing the Strength’ (RtS) schemes [1–4]. According to guidance issued by the Local Government Association, “the definition of high strength varies, but for the purposes of Reducing the Strength schemes has tended to refer to products from around 6.5 per cent ABV [alcohol-by-volume] upwards” ([5] p.6). These products are usually classified as strong beers and ciders sold at low prices. Super-strength lager is often sold as single 500 ml cans and well-known brands include Carlsberg’s Special Brew, Tennent’s Super, Kestrel Super and Skol Super. White cider is primarily sold in 500 ml cans, 1 litre or 3 litre plastic bottles and most available brands are 7.5 % ABV. Well-known brands include White Ace, Frosty Jacks and White Star.

At the time of the intervention described in the paper (2013–2014) a single 500 ml can of 9 % ABV beer contained more than the maximum daily alcohol intake for men (3–4 units) and women (2–3 units) recommended by UK health guidance [6]. A single 3 litre bottle of white cider also exceeded the recommended weekly alcohol intake (21 and 14 units for men and women, respectively) [6]. The UK Chief Medical Officer recently updated the alcohol guidelines, recommending that men and women consume no more than 14 units of alcohol per week, spread over at least three drinking days [7]. Super-strength products continue to be sold in quantities that exceed these recommendations.

Alcohol consumed in large quantities for prolonged periods is causally associated with both acute and long-term health problems [8–10]. Wider societal problems associated with super-strength alcohol include street drinking and homelessness, anti-social behaviour, underage drinking and family breakdown [11]. There is no evidence that super-strength alcohol has a unique or ‘special’ type of harm that would not be experienced from consuming the same units of alcohol in another form [12], rather it is the availability (convenience and branding) and low unit cost of these drinks that raise them as an issue of interest to public health practitioners [13]. One Australian study demonstrates a positive association between high-strength beer and cask wine consumption at a population level and alcohol-related criminal activity and alcohol-related morbidity [14]. There is evidence linking the price of alcoholic beverages and the volume consumed at a population level [15].

RtS schemes have become increasingly popular as a method to address the negative impact of super-strength products, and since 2012, approximately 80 schemes have been launched across England [16]. The intervention was originally launched in Ipswich, Suffolk, although Thames Reach, a large homeless charity, has been campaigning against super-strengths since 2005 [11]. RtS schemes vary in nature with regards to the specific drinks or populations targeted, but in general, local authorities ask local retailers licenced to sell alcohol for consumption off premise (such shops are called ‘off-licences’) in specific areas to voluntarily remove super-strength alcohol from sale [5].

RtS speaks to both the physical and economic aspects of availability [17]. If compliance is widespread, the intervention removes an entire group of products from an area, thereby reducing the quantity and variety of different types of alcohol available. Where super-strength beers and ciders represent the cheapest products on sale, the intervention will also raise the price of the cheapest available unit of alcohol in participating shops. Due to the relatively lower rate of alcohol levied on still ciders, white cider is almost universally the cheapest unit of alcohol available in shops. A study of heavy drinkers’ habits identified those who drank white cider as the population group consuming the most alcohol [18]. An organisation working with homeless and street drinkers identified super-strength lager as a preferred drink, and a cause of harm, amongst these groups [11].

Both UK and international health agencies recommend increasing the cost of alcohol to address alcohol-related harms, and evidence suggests that higher alcohol prices will most affect those who drink at harmful levels [19–21]. Off-licences, primarily small independent retailers, as opposed to supermarkets, have been found to sell 95 % of all alcohol consumed by heavy drinkers in Scotland. White cider was found to be exclusively available at these outlets [13]. Voluntary bans on super-strength products in Portsmouth have reported high levels of shop compliance with the intervention [5] and Ipswich, Suffolk has reported a reduction in crime and anti-social behaviour [22], although to date no robust evaluations have been published.

In this paper, we address the question of whether a targeted, voluntary approach to reducing alcohol availability can achieve the prerequisite of successfully enlisting retailers to volunteer. We also present early data on the effect an RtS scheme may have on the cheapest available alcohol. This is directly relevant to national and international debates over the relative merits of voluntary and compulsory approaches for reducing alcohol availability, as well as debates over local verses national level interventions [23–25]. It also has parallels with other interventions aimed at restricting particular alcohol products, such as restrictions on the sale of cask wine in parts of Australia [26, 27].

In 2013–2014 RtS schemes were implemented in the London Boroughs of Islington and Camden. In a related paper (currently submitted) we explore responses to RtS from the perspective of target populations of drinkers and front line staff who work with them. We have also conducted a separate quantitative study of RtS’s impact on alcohol sales using retail data (also currently submitted). The aim of this study is to evaluate the Islington and Camden schemes by assessing the effect on the cheapest available unit of alcohol in off-licences and retailers' willingness to participate. Using quantitative and qualitative process data, it explores whether a voluntary reduction in alcohol availability through this intervention is feasible, what influences retailers’ choice to participate or not, and how retailers believe their participation will influence alcohol purchasing amongst the targeted population.

## Methods

As part of a mixed methods evaluation we present data from local authority audits of off-licences and qualitative findings from interviews with retailers. The evaluation was conducted by members of Islington and Camden’s joint public health team in collaboration with independently funded researchers from London School of Hygiene & Tropical Medicine (LSHTM). Local authority auditing processes did not require ethical approval. The LSHTM team obtained ethical approval through the London School of Hygiene & Tropical Medicine Ethics Committee.

### Intervention

In 2013–2014, voluntary RtS projects were introduced in areas considered to have street drinking and alcohol-related anti-social behaviour problems within two neighbouring London local authorities, Islington and Camden. The Islington and Camden intervention areas contained 63 and 78 off-licences, respectively. Each local authority implemented RtS independently by recruiting alcohol retailers to voluntarily remove super-strength beers and ciders (defined as cheaply sold drinks with ≥6.5 ABV) from sale in their shops with a view to reducing health and social harms associated with street drinking, but also recognising potential benefits to wider populations.

The projects were designed and implemented by licensing teams in partnership with police and public health practitioners. Repeated visits were made to local premises to advocate for voluntary participation on grounds of social responsibility. Letters and visits to off-licences by the licensing and licensing police teams were used to raise the profile of the scheme. In addition, in Camden a launch event was organised by the council, which was attended by public health, the business improvement district and the local media.

### Sampling

The local authorities held data on all local off-licences; all stores in the intervention areas were included in the audit to provide data on compliance with RtS. Shops known to be selling super-strength alcohol pre-intervention were included in the qualitative fieldwork. Stores were sampled for qualitative fieldwork based on the assumption that around 40 of the 78 stores selling super-strength would be sufficient to provide a purposive sample that covered different geographical areas, shop types and shops that participated or declined to participate in RtS.

Sampled stores were visited to obtain consent to participate in the study from licence holders, managers or staff (which we refer to collectively as ‘retailers’). Retailers that did not participate in RtS were over-sampled to ensure this group was well represented. Visits took place between 3 and 6 months after intervention implementation commenced. Two researchers conducted each visit from a pool of five researchers. All interviewers were professionals with experience of conducing qualitative research.

### Data collection

Data on sign-up and adherence was provided by local licensing teams who made regular visits to off-licences to record the prices of alcoholic beverages and to audit whether ≥6.5 ABV beers and ciders were on sale. Where possible, public health practitioners objectively assessed the unit cost of alcohol in RtS participating shops. Pre-intervention prices were obtained from shop managers or shop staff and relied on their recall of the product prices. The price, container size and brand of the cheapest beer, cider wine and spirits available in both individual and multi-buy deals was recorded on a pro-forma during visit. Where a drink type was not available this section was left blank. Where the cheapest unit of any drink type was not clear, data on several cheap products was collected for later calculation and comparison.

A topic guide was developed to enable interviews to explore retailers’ views on the scheme, reasons for (not) participating in RtS, and views on how the scheme impacts on purchasing amongst the target population as well as broader impacts on the retailer and community. This guide was developed in collaboration with the licensing team who implemented the schemes and was designed to elucidate the motivations for participation, as well as the barriers. The guide provided a starting point for discussion but there was also sufficient scope within the interview for the participants to guide the discussion. The main themes in the topic guide were: knowledge of RtS, rationale for (non-) participation, impact on alcohol availability, challenges of participating in the scheme, impact on the shop, response from super-strength drinkers, intervention sustainability and suggestions to improve the intervention. Participant responses were recorded in writing during the interviews on a pro-forma with space for verbatim quotes. To aid recruitment, interviews were conducted in the shops as this was convenient for participants. However, we did not regard shops as a suitable location to audio-record the interviews due to the presence of customers.

Due to the short time span between implementation and evaluation no efforts were made to assess the wider impact of the scheme on levels of drinking, health harms or anti-social behaviour.

### Analysis

The cheapest unit of alcohol available, regardless of drink type, was calculated for each individual off-licence using the price, container size and ABV data. The median cheapest unit was then calculated across the entire intervention area and this data was plotted in terms of median and inter-quartile range pre- and post-RtS. A sub-analysis was conducted on only those shops that participated in RtS in order to understand the potential effect if the intervention achieved universal sign-up.

Post-visit interview notes were written up and reviewed by two researchers to draw out common themes. These were reviewed by a third author and through discussion amongst the research team, we drew out the shopkeepers’ prevalent and divergent opinions about RtS.

## Results

Forty-three off-licences were approached for interview and 39 (91 %) agreed to take part; 20 out of 24 Islington shops and all 19 Camden shops approached participated in the interviews. Nine interviews were from shops that did not agree to participate in the RtS scheme. Interviews were held with staff in managerial and sales positions as Licencees or Designated Premise Supervisors were often not available.

### Impact on the availability of super-strength

Prior to the intervention, 74 % (*n* = 47) and 39 % (*n* = 31) off-licences sold super-strength in Islington and Camden, respectively (Table 1). During the 3-6 month period following the intervention launch, 33 % (*n* = 21) of off-licences in Islington and 5 % (*n* = 4) of off-licences in Camden continued to stock these products. There remained variation within boroughs with sign-up tending to cluster geographically. There was an observed positive effect of neighbouring off-licences signing up and vice-versa.

**Table 1:** Availability of super-strength alcohol in the intervention areas (Pre- and Post-RtS)

	Number of off-licences in area	Super-strength available pre-intervention	Super-strength available post-intervention^a^	Relative reduction^b^
Islington	63	47 (74 %)	21 (33 %)	41 %
Camden	78	31 (39 %)	4 (5 %)	34 %
Total	141	78 (55 %)	25 (18 %)	37 %

^a^Visits conducted between 3 and 6 months after initial retailer sign-up to RtS scheme

^b^Relative reduction in the proportion of off-licences selling super-strength in each area

### Impact on the affordability of alcohol

**Fig. 1:**
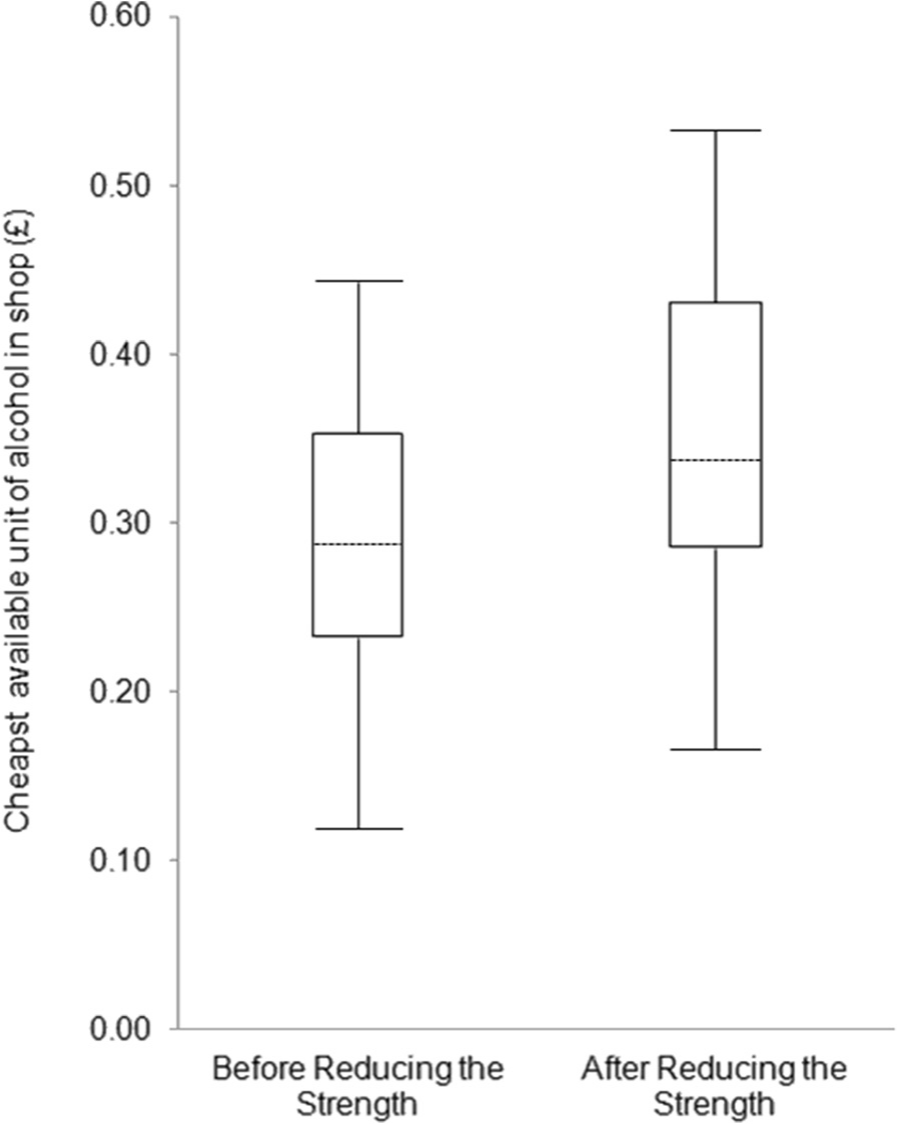
Cheapest available unit of alcohol in shops in the intervention area (*n* = 33). Median, quartiles and range showing price of cheapest unit of alcohol available in Islington and Camden off-licences before and after participation in Reducing the Strength

Price data was included for 33 of 39 shops visited. White cider, where available, was found to be the cheapest unit of alcohol available pre-intervention with prices as low as £0.12 per unit. Super-strength lager was found to be more expensive with the cheapest available unit across all shops costing £0.22 in a multi-buy offer. In a minority of cases the cheapest unit of alcohol identified was not classified as super-strength. In particular, multi-buy offers of cider with < 6.5 % ABV were found that provided the cheapest available unit of alcohol. Despite this there was an overall rise in the median price of the cheapest unit of alcohol from £0.29 to £0.33 (Fig. 1) available across the entire intervention area. The absolute cheapest available unit rose only slightly due to non-participation of some shops selling white cider. There was an increase in the cheapest alcohol unit available in 17 of the 33 shops surveyed (52 %). Of the shops that took part in RtS (*n* = 22), 85 % saw an increase in the cheapest available unit and the median cheapest available unit across all participating shops rose from £0.33 to £0.43.

### Views of shop managers and staff

#### Rationale for participation in RtS scheme

Overall, interviewees demonstrated a reasonable level of understanding of the immediate aim of the projects – to remove from sale cheap alcohol of ABV 6.5 % or above – and the further aim of reducing street drinking. Addressing health concerns associated with excessive alcohol consumption was recognised to a lesser extent. Some participants identified closely with the aims of the project, recognising street drinking as a problem in the area and at times considering their own perceived responsibility in enabling such activities:

*A lot of anti-social behaviour to reduce it which affects us…we had a lot of trouble and the neighbourhood was not happy* [Pilot participant, Camden]

Concern about the “*neighbourhood”* being unhappy illustrates how retailers could see attempts to reduce street drinking as a way of appeasing a wider customer base of local residents. Other retailers hoped participation in RtS would reduce anti-social behaviour within their own shops, linking street drinkers with problems such as shoplifting, verbal abuse and at times physical abuse of staff:

*It seemed like a good thing to do. They [street drinkers] were causing me problems… getting abusive* [Pilot participant, Islington]

More typically, reasons for joining RtS centred on a desire to co-operate with the licensing authorities. Interviewees mentioned wanting to “keep the council happy” by participating. There were differences in understanding with regard to the voluntary nature of the intervention, even though non-participating stores continued to operate in their area. Some retailers described a decision to participate taking the form of a voluntary agreement to new licensing conditions which would then be enforceable. Across the sample, different perspectives of what constituted ‘voluntary’ emerged, with some believing that the intervention was not voluntary:

*We thought if we don’t do it, we’ll lose our licence – this is our bread and butter* [Pilot participant, Islington]

#### Rationale for non-participation in RtS scheme

From the shops that had not participated in RtS, interviewees consistently justified non-participation in terms of perceived financial impact. These interviewees explained that they were concerned not only by the loss of trade from the removed super-strength items, but also by additional items that some customers buying these products also purchased:

*At first, I thought, ‘why not?’ I like to be good with the council [but] as a small business I have to look out for my cost…. You realise that people don’t just buy one thing, it’s a package… beer, cigarettes, paper, and so you lose out on that money too* [Non-participant, Islington]

The voluntary nature of the approach – and the knowledge that other retailers in close proximity had not signed up to the pilot – reinforced the view among these interviewees that participation was not a financially viable option within the difficult financial climate they described operating within. The increase in the number of local supermarkets and raising business rates and fees were frequently referred to. These issues were compounded by the prospect of competition from shops that were not participating in RtS:

*It’s not just about us doing it. If I sign up and next door doesn’t they are just going to go there.* [Non-participant, Islington]

Interviewees described the need for an even playing field, with super-strength products removed from sale from all retailers – at a pan-London or national level – before they could further consider participation in the pilot. In particular, there was a perception that supermarkets had not been included within the pilot with interviewees from smaller, independent shops citing their participation as vital before they could consider signing up themselves. However, these interviewees questioned the likelihood that this degree of sign-up could be achieved on a voluntary basis and reported that they believed nothing short of a ban would be successful in achieving this aim:

*They should just ban the drinks … That way, they wouldn’t go to other shops. Those who drink will always find a way anyway. The only way to stop street drinking is to ban alcohol.* [Non-participant, Islington]

Interviews with non-participants in the scheme included representatives from two national supermarkets. Despite the fact that one of these stores had initially signed up to the scheme, both reported that at an individual store level, managers had little to no discretion over the product lines stocked. The researchers were informed that in order to remove super-strength beers and ciders, this would need to be sanctioned by their respective head offices and rolled out on a national basis. One interviewee gave the rationale for this as the importance of consistency; that customers could walk into any store around the country and be able to purchase a similar range of items. The other offered a more multi-faceted explanation, elaborating on the potentially damaging impact that such a move could have on the relationships with the major breweries, even though the manager claimed that sales of super-strength were low:

*It can be part of the deal with the breweries. So in order for us to stock the standard Carlsberg lager we have to carry Special Brew, even if we don’t sell much of it.* [Non-participant, Islington]

#### Perceived impacts since joining scheme

A few participants felt that there had been a reduction in levels of street drinking, violence and litter in the area surrounding their premises. They attributed this to anti-social customers going elsewhere to shop for their super-strength. Some described ambivalent feelings trading the benefits of reduced anti-social behaviour within their shop against loss of sales:

*In one way it’s good because you get more decent customers but you also lose trade* [Pilot participant, Islington]

There was a widely held belief among interviewees that the majority of customers were now going elsewhere to buy super-strength beers and ciders. Interviewees highlighted that the voluntary, and targeted approach of the pilot meant that customers looking for super-strength rarely had to walk more than a few minutes before being able to purchase this: either to a shop within the pilot area that had opted not to participate, or to one which falls just outside the boundaries of that particular pilot area. Some independent shopkeepers spoke of witnessing regular customers who consumed super-strength switching to nearby rival independent stores that were not participating in RtS:

*We’ve lost business – we see people buying them [super-strength beers and ciders] from other shops* [Pilot participant, Islington]

*Other customers are going elsewhere. I see them.* [Pilot participant, Islington]

Some participants gave rough estimates of the percentage of their alcohol trade affected by RtS, ranging from 5 – 20 %. In other cases, participants reported customers substituting alcohol products, sometimes by stealing more expensive drinks, but more typically by buying lower ABV products. Shops tried to encourage this form of substitution through promotion of lower ABV beers and ciders:

*We’re trying to push lower [ABV] beers and we’re getting close to making up sales.* [Pilot participant, Islington]

## Discussion

This study is one part of a multi-methods and multi-site evaluation of RtS. It has been designed to produce early evidence on the feasibility of an intervention that relies on voluntary participation from shops. We found relatively high rates of participation by off-licences in the intervention areas and evidence that across the intervention area the median price of the cheapest unit of alcohol available increased. The rise was relatively small, although the target population of homeless and street drinkers have few financial resources making them susceptible to relatively small changes in economic availability [28]. The qualitative findings suggest that even a relatively small minority of non-participating shops can potentially deter voluntary compliance with the intervention and undermine its impacts if customers find it easy to access shops where they can still buy super-strength products. The relatively small geographical implementation area and voluntary nature of the intervention make this substitution of shops viable. The localised and voluntary nature of the intervention also presents other challenges for implementers. For instance, our findings suggest that larger retail chains make decisions to participate at a regional or national level, which means that local authorities wanting to implement RtS may be obliged to negotiate with the head offices of multiple national supermarket chains to ensure their participation.

The fact that RtS is a local-level intervention, generally delivered on a small scale, also has implications on the kinds of research approaches that are feasible and useful in this context. The local-level delivery means that the number of shops involved and the drinking population targeted were small, making sufficiently powered quantitative analysis difficult. In this paper we have included some basic quantitative data on shop uptake and minimum prices but rely on qualitative findings based on a purposive sample of half the total ‘population’ of off-licences known to have sold super-strength prior to the intervention.

The research also represents collaboration between local practitioners and academic researchers intended to maximise the utility of the study as a resource for informing practice. One requirement to achieve this was that the study provided timely findings about outcomes that local practitioners could plausibly seek to influence, such as intervention uptake and compliance [29–31]. Lengthy academic timescales have been described in previous literature as a barrier to evidence informed decision making if findings are reported too late to affect decisions driven by political timescales [32, 33].

It is recognised that public health researchers generally value evaluations with robust study designs that include greater numbers of participants to provide adequate statistical power for measuring health outcomes, preferably compared with a suitable control group [34, 35]. We too value such studies but we still argue the case for evaluations that cater specifically for the more immediate needs of decision-makers. Guidance on the evaluation of complex interventions recommends such studies to explore the feasibility of interventions and help inform decisions about whether larger scale intervention and evaluation are justified, so we would argue that our approach conforms to accepted standards of good evaluation practice [35].

### Limitations

Within the timescales of the evaluation we are unable to demonstrate any longer term outcomes such as reduced crime, anti-social behaviour, acute health harms or improved long-term health. Follow-up visits were not made to all off-licences as part of the evaluation and sampling was purposive rather than random. This research should be viewed as a ‘snapshot’ over a relatively small period of time rather than as the final and continuing result of the intervention.

The interviews were conducted by local authority staff. It is possible that this would influence the potential for interviewer bias, if (for example) participants decided that it was in their interests to emphasise their compliance and enthusiasm for RtS. Non-participation was low but interviewees were able to refuse for any reason they chose. We do not rule this bias out but we do highlight that many responses quoted in this article identified perceived problems with the intervention, and included some participants who described their refusal to participate in the intervention.

The involvement of independently funded academic researchers is intended to safeguard against the conflict of interest inherent in a local authority evaluating its intervention. However, whilst this form of ‘co-production of evidence’ between practitioners and academics is currently advocated amongst researchers, practitioners and grant holding bodies, it raises questions about the extent to which academic researchers involved can justifiably describe themselves as ‘independent’.

As many of the off-licences were small independent shops, the only member of staff available for interview was often serving customers at the same time the interview was being conducted, which meant that interviews were necessarily kept short. As a result, price data was not collected for all shops. In addition, prices of super-strength alcohol pre-intervention relied on the recall and accurate reporting of the interview. We believe this pragmatic approach helped to keep response rates high but at the expense of a richer dataset. Interviews were conducted in English, which shop staff could speak but not always as a first language. In the absence of audio-recoding, we are reliant on interviewers’ written fieldnotes. Participants were not contacted to verify these notes.

### Policy implications

Although voluntary and community initiatives are often small scale and may have less impact than more comprehensive policy interventions, they are sometimes seen as useful for tackling specific local problems particularly in contexts where resources are limited [36]. Examples of such interventions include the Alcohol Linking Program [37], the Queensland Safety Action Project [38] and the Swedish Stockholm Prevents Alcohol and Drug Problems (STAD) initiative [39].

Reducing the Strength projects are a clear example of an innovative local solution to a national problem and over 80 local authorities have implemented RtS schemes in the absence of a national minimal unit price (MUP). Prior to the introduction of RtS, a litre of 7.5 % ABV white cider retailed for around £1.50 in Islington and Camden, which is 20p per unit of alcohol. In England the 40p MUP proposed by the Coalition government in 2012 would have delivered a minimum price of £3.00 per litre of 7.5 ABV% beer or cider [40, 41]. An alternative alcohol pricing policy came into force in May 2014 banning the sale of alcohol below the total cost of duty and VAT combined [42]. This effectively introduced a MUP that varies by drink as duty differs substantially between alcohol types. This established a minimum price for one litre of 7.5 % ABV white cider of 48p or 6.4p per unit of alcohol [40, 43], a price far below the current cost and even further below the proposed MUP from 2012. The policy did have some effect on the minimum price of a super-strength lager establishing a minimum price of £1.30 per can [40, 43], higher than the price in some Islington and Camden off-licences during the research (pre-legislation). Research modelling concluded that a 40p to 50p MUP would result in 40 to 50 times greater effect on consumption than the floor price approach [40].

From the local authority’s perspective, persuading retailers to voluntarily participate in RtS represents an extremely resource intensive way of achieving outcomes that could be potentially derived from a national MUP policy. RtS focuses on the complete removal of a narrow range of products, primarily super-strength beers and white ciders, whereas a MUP would allow consumers to buy these products, but at a price linked to their alcoholic content, and hence likely level of harm, rather than their tax regime. In contrast, voluntary schemes such as RtS are inherently susceptible to the problem that retailers stand to potentially benefit by not complying if they attract customers away from compliant shops.

The perceived merits and limitations of voluntary alcohol interventions such as those involving alcohol retailers have been widely debated in the academic literature and policy circles [36]. The research literature on interventions to reduce population-level alcohol harms provides evidence that mandatory rather than voluntary approaches are more likely to be effective [44]. Moodie et al. concluded in 2013 that despite common reliance on industry self-regulation and public-private partnerships in policy, there is no evidence of their effectiveness [45]. Babor has argued that voluntary codes are subject to under-interpretation, under-enforcement and poor compliance [8]. A review of voluntary UK social responsibility measures found poor compliance and interventions that were judged to be not fit for purpose [46]. A study of Australia’s voluntary labelling scheme found the labels were difficult to understand and did not have the desired health impact [47].

The alcohol industry has stated its interest in what is sometimes called responsible retailing. Heineken, for example delisted two high strength ciders, White Lightning and Strongbow Black, in 2008 citing recognition of the links between the product and social harms following a visit to an AddAction project [48]. The UK’s Public Health Responsibility Deal, a public-private partnership where industry and government actors sign up to pledges aimed at improving public health [49], included a pledge to reduce the total alcohol in a single serving carbonated drink (e.g. a can of lager or cider) to less than the maximum recommended daily intake for an adults. The manufacturers of some super-strength drinks have signed up to this pledge [50]. In some cases, this has resulted in a reduction of can sizes to 450 ml, although the products have not been removed from sale. Changes under this Responsibility Deal were evaluated as being unlikely to contribute significantly to reductions in alcohol consumption [49].

Our findings suggest that there is some support amongst retailers for a more interventionist approach on alcohol sales, echoing evidence from a previously published cross-sectional survey of small retailers in Scotland, which found support for another regulatory intervention, MUP [51]. These findings provide a reminder that regulation need not necessarily take place against the perceived interests of the private sector (or parts of it, assuming the private sector is heterogeneous), and it is possible that some private sector stakeholders view regulation as a fairer and economically less risky option than voluntary participation in schemes like RtS. Our findings also suggest that voluntary interventions can be perceived in different ways, with some shop keepers exercising their right not to participate in RtS, others apparently supporting the intervention, whilst others gave a more pragmatic view that participation could help them maintain good relationships with local authorities and so safeguard their business against unspecified future actions from the licensing authorities. Hence, we see that the conceptual boundaries between voluntary and mandatory action begins to look more fluid and subjective when viewed at close quarters in relation to this intervention. We speculate that within mandatory frameworks there may be points at which there is a choice, and we suggest that within voluntary frameworks the available choices may be weighted by understandings or perceptions of potential costs and benefits.

## Conclusions

The RtS interventions studied here have led to the majority of off-licences within the intervention areas removing super-strength from their shelves. Retailers remain convinced that customers often switch to non-participating shops to continue to buy these products. This illustrates the limitations of local, voluntary approaches to reducing alcohol availability as part of harm prevention strategies, even when the intervention is well delivered and achieves high rates of compliance. Even some of the retailers who refused to participate in RtS support compulsory measures which, they believe, would help them avoid negative financial impacts.

## Abbreviations

ABV, alcohol-by-volume; LSHTM, London School of Hygiene & Tropical Medicine; MUP, minimum unit price; RtS, Reducing the Strength; UK, United Kingdom.
